# Women's Health Information Survey: Common Health Concerns and Trusted Sources of Health Information Among Different Populations of Female Patients

**DOI:** 10.1089/whr.2020.0118

**Published:** 2021-06-01

**Authors:** Casey Dluhos-Sebesto, Trisha E. Jethwa, Tais G.O. Bertasi, Raphael A.O. Bertasi, Livia Y. Maruoka Nishi, Sally Ann L. Pantin, Sandra L. Argenio, Ali Shahsamand, Adeyimika Omololu, George G.A. Pujalte

**Affiliations:** Department of Family Medicine, Mayo Clinic, Jacksonville, Florida, USA.

**Keywords:** access to information, health resources, mental health, primary health care, surveys and questionnaires, women's health

## Abstract

***Background:*** Women are more likely to search for information on behalf of both themselves and others, thus making them a valuable target for health information research. The purpose of this project was to identify and compare the most important medical concerns and sources of health information trusted by women in relationship to demographic differences in two different clinical settings within the same geographic area (Mayo Clinic Department of Family Medicine and Beaches Community Health care—a Sulzbacher Center Clinic).

***Methods:*** A novel survey tool was developed to obtain information regarding the age, race, and socioeconomic demographics of patients as well as the patients' personal significant medical concerns and trusted sources of health information.

***Results:*** Despite the huge development of health care information delivery through online resources, in our study, the majority of patients from both clinics still used and viewed their primary care provider as the most trusted source of health information. The health concerns most reported by both populations included cardiac health, breast and other cancers, and obesity; meanwhile, mental health was significantly more reported by patients from the free clinic.

***Conclusions:*** Education level may be an important factor of the awareness and ultimate treatment and prevention of these prevalent diseases. Furthermore, our study results may help improve patient satisfaction, knowledge, and health outcomes.

## Introduction

Research has shown that women tend to seek out health care information more than men.^1^ Women are also more likely to consult a greater variety of resources, including friends, family, and alternative health professionals. In addition, women are more likely to search for information for both themselves and on behalf of others.^2^ This suggests that the traditional gender role of women as primary coordinators of their family's health care, parenting, and caregiver responsibilities contributes to their tendency to seek health care information of high depth and scope from various sources.^3,4^

In 2005, Hesse et al.^5^ determined that, while the most trusted source of health care information was provided by physicians, compared to the internet, women are more inclined to place increased trust in online sources. Around the same time, Sillence et al.^6^ also determined that the use and trust of less-regulated websites (*e.g*., personalized or retail oriented) were already on the rise. With the accessibility of the internet becoming widespread and online media usage gaining an important role in society through technologies such as smartphones, it is no surprise that 75% of Americans turn to the internet as a source of health care information.^7,8^ Moreover, currently, the internet is the most frequently used health care information resource for 80% of women.^1^

What remains unknown is the extent to which the trust dynamics have continued to shift over the past decade from the physician's office to online or social resources, particularly in special groups like underprivileged women with less access to health care professionals as well as younger women who have higher rates of internet use. Sedrak et al. assessed the online health information-seeking behavior among 72,806 women 65 years of age and older with chronic illnesses. Women who used the internet to obtain health information were relatively younger (median age: 76 vs. 81 years), earned a higher income, and achieved a higher educational level.^9^

Another recent study assessing the influence of emotional states and education levels to the use of online resources for health information showed that college educated and people who reported being sad some or all of the time, and those who are anxious most of the time were significantly more likely to seek online health information.^10^

Rowley et al.^2^ determined gender to be the most important influencer when seeking online health information, in addition to trust behavior. The authors^2^ called for further exploration of relationships between other demographic variables as they pertain to health information and trust, particularly age and stage of life. In addition, Hallyburton and Evarts^1^ showed that college-age students are generally ambivalent regarding the credibility of online health resources, indicating that little is known about the influence of age on trust. Similarly, although Lin et al.^7^ showed that the digital divide is narrowing in terms of internet use, little research is available concerning socioeconomic factors and trust behaviors regarding particular health information resources.

In seeking to build upon and answer some of the questions posed by the research above, the purpose of this project was to identify and compare the most important medical concerns and health information sources trusted by women in relationship to generational and demographic differences in two drastically different clinical settings within the same geographic area. While a growing body of research has focused on improving the delivery of health care information and communication through the development of high-quality online and interpersonal resources for the masses, our focus is centered on the patient, for whom health care providers are most likely to have an impact.

Our intention is that the information in this study may offer health care providers the opportunity to streamline the communication of health care information by developing educational materials and protocols and to improve patient satisfaction, knowledge, and health outcomes. We chose to focus on women in our study, given that they are more likely to seek medical information for themselves and others. However, this survey could ideally be adapted for use in any clinical setting across all genders, languages, and countries. Within the overarching theme of this study, several hypotheses were tested:

(1) Underprivileged female patients at free clinics are more likely than those seen at private payer clinics to use and trust sources of health care information, such as the internet, friends, family, religious leaders, and media. There are indications that this group has difficulty accessing medical care and may have education levels making them more likely to believe health information on the internet.^11,12^
(2) Underprivileged patients are more likely to have poorer self-reported quality of health and health literacy than private payer patients.
(3) Private payer patients are more likely to be concerned about preventative health issues.
(4) Patients older than 60 years are more likely than younger patients (younger than 40 years) to be concerned about chronic health conditions. (*e.g*., diabetes, hypertension, cancer, *etc*.)

## Methods

This study was approved by the Mayo Clinic Institutional Review Board (IRB; 12-008925). A novel survey tool was developed to obtain information regarding patient age, race, and socioeconomic demographics, as well as important medical concerns and trusted sources of health information as they relate to them personally. The survey tool was developed by first listing questions that were thought by the authors as pertinent to finding out how women look for health information, and the characteristics of those women. The draft questionnaire was then improved upon by the questionnaire development team in Mayo Clinic Florida. The resulting first version was tested on female learners, residents, and patients, intermittently.

Based on input and observations on ease of understanding and answering the questions, the final version was developed. The 36-question survey specifically asked patients to rank the usefulness and trustworthiness of the following health information resources: primary care provider (PCP), alternative health specialist (clinicians primarily employing manual therapies, alternative medicinal systems, traditional Asian medical systems, and mind-body therapies, beyond what is offered in Western, evidence-driven and based medicine), family and friends, religious institutions, print material, TV and radio, and internet. Other variables included insurance status, self-rated quality of health, and self-perception of health literacy.

The survey was administered to female patients 16 years of age and older. These patients attended appointments over a 4-week period in 2014 at two clinic locations in Jacksonville, Florida: The Mayo Clinic Department of Family Medicine (MCDFM) and Beaches Community Health care—a Sulzbacher Center Clinic (BCH). The paper survey and a written consent form were presented to each eligible patient by reception staff upon check-in for their appointment and collected by members of the research team. Exclusion criteria included all male patients and patients younger than 16 years.

MCDFM is a primary care center with professional care provided by 18 family medicine residents, 9 attending physicians, and 2 advanced registered nurse practitioners. The payer mix is varied, with 47% having commercial insurance products, 30% Medicare, 18% Health Maintenance Organization or Preferred Provider Organization plans, and 2% a US Department of Defense Military Health System program. Of its 9,240 patients, 57% were women and 43% were men.

BCH is a federally qualified health center and Federal Tort Claims Act-monitored facility that offers a complete range of health care services to 6,260 patients. Professional medical care is provided by two physicians and four midlevel providers, who consist of either advanced registered nurse practitioners or physician assistants. Regarding their payer mix, close to 87% of their patients are uninsured, 11% of their patients are covered by Medicaid or other public insurance, and less than 2% are covered by Medicare.

Descriptive statistics for the survey answers were reported as frequencies and percentages, while patient age was reported as a mean (standard deviation: SD). The answers were compared according to the clinic location (MCDFM or BCH) using the Fisher's exact test. Statistical analysis was performed with SPSS (version 1.0.0.1347) for Mac OS. All statistical tests were two sided with the alpha level set at 0.05 for statistical significance.

## Results

The overall survey response rate was *N* = 144 for MCDFM and *N* = 52 for BCH. The demographic characteristics of patients are showed in Table 1. The age of respondents at MCDFM was significantly higher compared to BCH, with means of 40.9 ± 18.2 (mean ± SD) and 29.7 ± 10.9, respectively (*p* < 0.001). Women from BCH had significantly lower levels of education, with high school or equivalent education (*n* = 20, 41.7%) being the most common response. The second most common response was 1–3 years of college (*n* = 17, 35.4%). In the MCDFM group, 48 (35.3%) women completed 1–3 years of college and 43 (31.6%) completed 4 years of college (*p* < 0.001). Moreover, more patients from MCDFM were married (58.8% vs. 12.5%; *p* < 0.001).

**Table 1.** Patients Demographics, Socioeconomic Status, and Educational Level

	MCDFM (*n* = 144)	BCH (*n* = 52)
Age		
16–39	40 (30.1)	30 (66.7)
40–59	63 (47.4)	6 (13.3)
60–79	8 (6.0)	0 (0)
80+	22 (16.5)	9 (20.0)
Highest level of education completed		
Less than high school	1 (0.7)	3 (6.3)
High school or equivalent	20 (14.7)	20 (41.7)
1–3 years of college	48 (35.3)	17 (35.4)
4 years of college	43 (31.6)	3 (6.3)
Professional degree	12 (8.8)	2 (4.2)
Master's degree	12 (8.8)	3 (6.3)
Marriage status		
Single	20 (14.7)	19 (39.6)
Married	80 (58.8)	6 (12.5)
Separated	2 (1.5)	4 (8.3)
Divorced	14 (10.3)	18 (37.5)
Widowed	17 (12.5)	1 (2.1)
Domestic partner	3 (2.2)	0 (0)
Employment rate	78 (56.9)	19 (37.3)
Insurance coverage	135 (99.3)	1 (2)

BCH, Beaches Community Health care—a Sulzbacher Center Clinic; MCDFM, Mayo Clinic Department of Family Medicine.

Among the MCDFM patients, 78 (56.9%) of MCDFM patients were employed compared to 19 (37.3%) of BCH patients (*p* = 0.016). There were 135 (99.3%) women at MCDFM who had health insurance compared to only 1 (2%) at BCH (*p* < 0.001). The majority of MCDFM patients felt they had good (*n* = 48, 33.3%), very good (*n* = 5, 34.7%), or excellent (*n* = 21, 14.5%) quality of health, whereas the majority of BCH patients were within fair (*n* = 14, 26.9%) and good (*n* = 21, 40.4%) quality of health. There were 130 (97.7%) MCDFM patients who revealed that they had a PCP compared to only 20 (38.5%) of the BCH patients (*p* < 0.001).

**FIG. 1.**
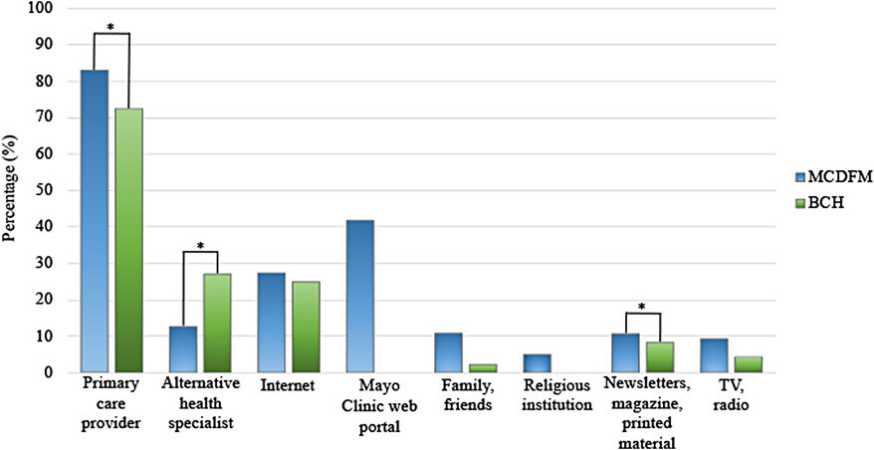
Source of Health Information. Percentage of participants who chose 4 or 5 on a scale of 1 to 5 (1 = never and 5 = always) when asked where they get most of their medical information. *p < 0.05. BCH, Beaches Community Health care—a Sulzbacher Center Clinic; MCDFM, Mayo Clinic Department of Family Medicine.

Across both locations, the most utilized source of information was the PCP, followed by alternative health specialist for BCH, and the Mayo Clinic Web Portal for MCDFM (Fig. 1). However, patients from MCDFM retrieved health care information more often from their PCP (83% vs. 72.5%; *p* = 0.03), news and magazines (10.7% vs. 8.3%; *p* = 0.03), and the Mayo Clinic Web Portal (41.7% vs. 0%; *p* < 0.001), while patients from BCH preferred alternative health specialists (27.1% vs. 12.6%; *p* = 0.006). No difference was found between the groups regarding the views of alternative family and friends, religious leaders, TV and radio, and internet as useful sources of information.

**FIG. 2.**
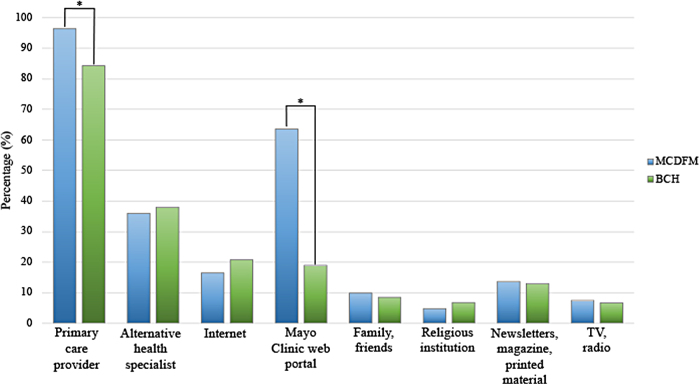
Trusted Source of Health Information. Percentage of participants who chose 4 or 5 on a scale of 1 to 5 (1 = least trusted and 5 = most trusted) when asked to rank how they trust the accuracy of information received by each source. *p < 0.05.

The same pattern of responses was found when patients were asked to rank how they trusted the accuracy of information received by each source (Fig. 2). Overall, for both groups, PCP was viewed as the most trusted source of information, followed by the alternative health specialists for BCH and the Mayo Clinic Web Portal for MCDFM. When comparing the groups, MCDFM patients placed more trust in their PCP (96.4% vs. 84.3%; *p* = 0.01) and the Mayo Clinic Web Portal (63.6% vs. 19%; *p* < 0.001) than the patients from BCH, while all other variables were not statistically different between them.

The health concerns reported most often by patients from both centers were cardiac health (28.5% at MCDFM and 23.1% at BCH) and breast cancer (27.1% at MCDFM and 32.7% at BCH). Significantly more patients from BCH reported mental health (17.3% vs. 5.6%; *p* = 0.01) and Pap smears (15.4% vs. 2.1%; *p* < 0.001) as a major concern. Osteoporosis was also reported significantly more within the MCDFM group (11.1% vs. 1.9%; *p* = 0.044). The commonly reported concerns per site are presented in Table 2.

**Table 2.** Most Commonly Reported Health Concerns by Site

Health concern	MCDFM (*n* = 144)	BCH (*n* = 52)
Cardiac health	41 (28.5)	12 (23.1)
Breast cancer	39 (27.1)	17 (32.7)
Other cancers	22 (15.3)	11 (21.2)
Obesity	23 (16.0)	7 (13.5)
Diabetes	12 (8.3)	9 (17.3)
Hypertension	12 (5.2)	7 (7.3)
Hyperlipidemia	5 (3.5)	3 (5.8)
Mental health^a^	8 (5.6)	9 (17.3)
Depression	2 (1.4)	3 (5.8)
Anxiety	4 (2.8)	2 (3.8)
Aging	14 (9.7)	1 (1.9)
Osteoporosis^a^	16 (11.1)	1 (1.9)
Menopause	7 (4.9)	5 (9.6)
Pap smear^a^	3 (2.1)	8 (15.4)
Nutrition	9 (6.3)	3 (5.8)
Fertility	6 (4.2)	1 (1.9)
Other	38 (26.4)	17 (32.7)

Data reported as *N* (%).

^a^ Statistically significant *p*-values (95% confidence interval): 0.01 (mental health), 0.044 (osteoporosis), <0.001 (pap smear).

## Discussion

Most of the women surveyed at MCDFM were married, while most at BCH were either single or divorced. Since 2000, lower levels of education and disadvantaged socioeconomic status have been associated with declining rates in marriage, staying married, and remarriage; meanwhile, highly educated white and black women have an increased likelihood of marriage, staying married, and remarriage.^13^ Supporting this finding, most participants at MCDFM had completed 1–3 years of college education, whereas most participants at BCH were high school graduates.

Higher levels of education likely allow for a larger salary and, therefore an increased likelihood of having insurance. In addition, most women surveyed at MCDFM were employed, while those surveyed at BCH were mainly unemployed, which is also likely due to their lower level of education.

Earlier studies have shown that compared to men, women are more likely to seek and use information from multiple resources simultaneously, including both health care providers and the internet, and they are also more likely to place trust in these resources.^1,4,5^ Also, women seek out other individuals, such as family and friends, as sources of health care information more often than men.^1,2^

Early research identified that older age, higher income, and higher education are associated with increased use of the internet for health-related information in women.^8,14–16^ This suggests that underprivileged groups who have less access to medical professional advice may also have less access or skills required to navigate internet resources and, thus, may tend to seek health care information from sources such as radio, TV, magazines, friends, family, church, and other social networks.

Alternatively, there is a growing body of literature that suggests that the gap is closing in terms of the digital divide in age and socioeconomic status,^1^ and minorities and disadvantaged groups who do use the internet to seek health information have improved health care delivery because of it.^7,17,18^ For example, uninsured patients are more likely to use the internet for health information than their insured counterparts.^14^

However, Lin et al.,^7^ in a study exploring racial and ethnic roles as they relate to health information-seeking behaviors and internet efficacy, failed to confirm the results of earlier studies; the authors detected only a marginal influence of race on these behaviors. Furthermore, their study identified high income as the only demographic variable with significant correlation to increased internet use for health site searches for information. They postulated that as increased internet diffusion through the pervasive use of devices like smartphones continues to close the gap in the digital divide, underserved populations with low income, lack of medical access, or minority status will use the internet to substitute or, hopefully, supplement consultation with health care professionals.^7^

Despite the present digital age with its easily accessible resources, the most trusted resource for health care information among our study populations was still the PCP; therefore, health care experts remain vital to health care information and decision making. This result refutes our theory that underprivileged female patients seen at a free clinic (BCH) are more likely than female patients seen at a private payer clinic (MCDFM) to use and trust other sources of health care information, such as the internet, friends and family, religious leaders, and the media. It also shows that the PCP continues to have a large impact on women's health care despite socioeconomic status.

In both studied populations, the internet was an important resource for health care information. This is consistent with the theory that the digital divide is closing as people of different socioeconomic statuses are experiencing better access to the same resources. The Pew Research Center^19^ reported that 61% of American adults look for health information online. This supports the finding in our study, in which the Mayo Clinic Web Portal and the internet overall were some of the most used and trusted sources of health information following direct information from a health specialist.

While most women seen at MCDFM mentioned frequently accessing the Mayo Clinic Web Portal, this was not a possibility for patients from BCH, but they still considered information from this portal accurate. The belief that some respondents trust on the Mayo website even though they had never seen it before may have more to do with belief in the brand “Mayo Clinic” itself instead of the content, which may indeed be the same as what could be found in similar health care provider websites. Overall, patient portal use is on the rise, especially in primary care clinics. However, the use of a patient portal is largely dependent on the infrastructure necessary for online delivery of health care,^20^ so finance- and resource-limited clinics like BCH are less likely to offer patient portal access.

Poor self-reported quality of health and poorer health literacy were more frequent among underprivileged patients than private payer patients. The majority of MCDFM patients felt they had good to very good quality of health, whereas BCH patients reported being more within fair to good quality of health. This could mean that lower health care literacy results in poorer health outcomes and, thereby, poorer reported quality of life.

As such, it is important for health care providers to find ways to rapidly assess health care literacy during visits. This information allows for materials and resources to be better directed to patients of different education levels, which is significant because it is crucial for patients to understand their diagnoses and treatment plans and be able to effectively communicate with their providers.

Although it was hypothesized that older women were more likely to be concerned than younger patients about chronic health conditions, such as diabetes, hypertension, and cancer, this was only proven to be true for diabetes and cancer. Another interesting finding was that significantly more women from the BCH group reported screening for cervical cancer (Pap smear) as an important health concern; this contrasted with our hypothesis that private payer patients would be more concerned about preventative health issues. This could be because, without insurance for larger procedures (private payers), patients with less financial means feel it is important to be preventative.

Our study showed that the majority of women surveyed at MCDFM felt cardiac disease was a major health concern, and rightfully so, as cardiovascular disease continues to be the leading cause of death among women in the United States, accounting for about one of every three deaths.^21^ However, it has been reported that this has not been historically well known by this population. In 1997, only 30% of American women surveyed were aware that cardiovascular disease was the leading cause of death in women; this increased to 54% in 2009 and subsequently plateaued when last surveyed in 2012.^21^ The uptrend in awareness may be secondary to efforts from the American Heart Association to increase women's knowledge of heart disease, such as the Go Red For Women campaign in 2003.

Despite these efforts, there is a continued gender gap in mortality rates from cardiovascular disease, with increasing rates in 35- to 44-year-old women. Furthermore, the incidence of myocardial infarction in African American women is higher in all age categories,^22^ and the death rate of African American women is reportedly 41% higher compared with white women.^23^ The strongest predictors for awareness in our data were education level, income level, and ethnic minority status. Thus, providing correct and reliable information can increase awareness, treatment, and control of diverse diseases.^24^

Our study also showed that breast cancer was a health concern for many women surveyed, especially within the BCH group, where breast cancer was the principal health concern reported. In 2019, it was estimated that among US women, there were 268,600 new cases of invasive breast cancer.^25^

There have been an increasing number of global health initiatives to address breast cancer, including efforts by Susan G. Komen for the Cure, the Breast Health Global Initiative, the US Centers for Disease Control and Prevention, the American Cancer Society, and the National Cancer Institute, as well as leading oncology societies across the globe. Despite these efforts, the infrastructure and resources for routine screening mammography are often unavailable in low- and middle-income countries.^26^ Fortunately, breast cancer mortality decreased by 40% between 1989 and 2017 due to improved breast cancer treatment and early detection.^25^

The results of this study further revealed that significantly more women within the surveyed MCDFM population felt osteoporosis was a health concern for them (11.1% vs. 1.9% for BCH). In a study of Australian women, bone mineral density testing, prior fracture after age 45, younger age, and lower self-reported general health were significantly associated with being very or somewhat concerned about osteoporosis and having a much higher or a slightly higher perception of osteoporosis and fracture risk.^27^

Poorer bone mineral density results were also associated with higher concerns and higher risk perceptions as well. In addition, the presence of comorbidities, having two or more falls in the preceding year, and maternal osteoporosis were associated with higher concern, while maternal osteoporosis, the presence of comorbidities, weight loss of five kg or more in the preceding year, and low body mass index were associated with higher perceptions of osteoporosis risk.^27^

Significantly more women within the BCH group reported mental health as an important health concern compared to MCDFM group. This could be associated with the fact that women often perform the traditional role of caregiver, with many domestic responsibilities. Also, women have added work responsibilities and generally higher stress levels, which likely lead to this health concern, especially for underprivileged women.^28^ According to the literature, older patients (age ≥55 years) may have less depression, anxiety, and self-stigma, and more social support compared to younger individuals,^29^ and considering that the population from MCDFM was significantly older than BCH group, generational differences may have also played an important role in this finding.

Our study has some limitations, such as its design and small sample size. Although interesting, our findings are speculative. They should not be extrapolated and must be analyzed with caution. We also could not determine the profile of race/ethnicity of our participants due to low number of responders to this question and we could not look into smaller groups by age. The larger groups were meant to accentuate differences in health information-seeking behavior, but future studies can look into smaller divisions of age groups to see if significant differences exist among women just 5–10 years apart in age, for example.

## Conclusion

Our study showed that despite the increasing development of the delivery of health care information through online resources, the majority of female patients are likely to consider their PCP to be their most useful and trusted source of health information. The top health concerns were different between the two clinics surveyed (cardiac disease at MCDFM vs. breast cancer at BCH), indicating that educational level may influence awareness and, ultimately, treatment and prevention of prevalent diseases. The results of this study may offer health care providers the opportunity to streamline the communication of health care information by developing educational materials and protocols, as well as improving patient satisfaction, knowledge, and health outcomes.

## Abbreviations Used

BCH: Beaches Community Health care—a Sulzbacher Center Clinic
MCDFM: Mayo Clinic Department of Family Medicine
SD: standard deviation
